# Diagnosis and treatment strategy of coronavirus disease 2019 with cardiovascular disease in elderly patients

**DOI:** 10.1002/agm2.12108

**Published:** 2020-05-14

**Authors:** Biao Cheng

**Affiliations:** ^1^ Department of Geriatric Cardiology Sichuan Academy of Medical Sciences and Sichuan Provincial People’s Hospital Chengdu China

31.2% of COVID‐19 patients are reported to be over 60 years old, 81% of the dead patients are elderly patients over 60 years old.^1^ Up to 24:00 January 22, 2020 there were 425 confirmed cases of COVID‐19 as reported by the National Health Commission of the People’s Republic of China.^1^ Furthermore, 162 (or 38.1%) of these patients were older than 65 years and 40% of this older age group were complicated with cardiovascular disease.

The mechanisms of heart injury caused by viral pneumonia are as follows^2^: the virus directly damages the myocardium, resulting in viral myocarditis; at the same time, viral infection leads to an inflammatory storm that causes cardiovascular events, such as acute coronary syndromes and arrhythmias. According to the research,^3^ these cardiovascular complications could persist for a long time, even for more than 10 years.

Even with advanced medical examination methods and powerful medical data analysis for reference, it is still very difficult to select diagnostic strategies for patients with pneumonia and cardiovascular diseases because the pulmonary imaging features of these two diseases are complicated. For example, ground‐glass shadow and septal thickening are shared features of the two diseases. Therefore, clinical experience is very important in such cases.

## 1 PROGRESS IN EARLY DIAGNOSIS OF COVID‐19 WITH CARDIOVASCULAR DISEASES IN ELDERLY PATIENTS

In China, the diagnosis of COVID‐19 goes through three stages. In the first stage, suspected cases are tested for the new coronavirus nucleic acid with real‐time fluorescence‐based polymerase chain reaction or virus‐gene sequencing is carried out. In the second stage, the clinical diagnosis is added, and pulmonary computed tomography images are checked for consistency with the appearance of COVID‐19 in suspected patients. The current diagnosis includes a positive viral nucleic acid test, highly homologous gene sequencing, and immunoglobulin positive (Ig)M/IgG antibody test.

Diastolic function is reduced in the elderly, especially those with hypertension, coronary heart disease, atrial fibrillation, and left ventricular hypertrophy, which can cause and/or exacerbate diastolic dysfunction. The 2016 European Society of Cardiology guidelines^4^ divide heart failure into reduced ejection fraction (HFrEF; ie, left ventricular ejection fraction [LVEF] < 40%), mid‐range ejection fraction (HFmrEF; ie, LVEF of 40%‐49%), and preserved ejection fraction (HFpEF; ie, LVEF ≥ 50%). These guidelines emphasize the importance of LVEF, ratio of peak early diastolic flow velocity (E) and peak atrial diastolic flow velocity (A) (E/A), and B type natriuretic peptide. The classification of HFpEF (or diastolic heart failure) confirms the European Heart Failure Association (HFA)‐PEFF rule established by the HFA in 2019,^5^ and a score ≥ 5 points indicates left ventricular diastolic heart failure. A study published in Circulation in 2018^6^ suggested an evaluation subsystem for chronic heart failure with preserved ejection fraction: The H_2_FPEF score ranges from 0 to 9 and differentiates HFpEF from noncardiac dyspnea. The diagnosis of ventricular premature beats requires Lown’s classification.

### 1.1 Problems 

At present, the supply of COVID‐19 nucleic acid testing kits varies greatly among different cities in China. Clinicians not specializing as cardiologists have little knowledge of cardiovascular diseases, and their clinical experience varies greatly.

## 2 EARLY RISK STRATIFICATION AND THE SIGNIFICANCE OF MULTIDISCIPLINARY TEAMWORK

Risk stratification in the early stage plays a great role in predicting prognosis and developing treatment strategies. As novel interventions are introduced constantly, risk stratification is also updated. Multiple individualized assessments are performed according to the specific conditions of patients and updated risk stratification. The purpose of early assessment and early risk stratification is to clarify the diagnosis and identify high‐risk patients for different treatment strategies.

COVID‐19 is classified into mild, moderate, severe, and critical cases according to the seventh edition.^7^

Cardiac function stratification:

1. Left ventricular diastolic dysfunction assessment (2019 HFA).^5^ Grade I: E/A ≤ 0.8, E peak ≤ 50 cm/s; Grade II: 0.8 < E/A ≤ 2; Grade III: E/A > 2.
2. Classification of chronic heart failure (HF) based on left ventricular ejection fraction (LVEF) values: HF with preserved ejection fraction (HFpEF), HF with mid‐range ejection fraction (HFmrEF), and HF with reduced ejection fraction (HFrEF).
3. Assessment of acute myocardial infarction (AMI): Killip classification based on HF (Grades I‐IV); Forrester classification according to hemodynamics, which can be used as a basis for evaluation and treatment.
4. Classification of acute heart failure: According to the presence of hyperemia and/or hypoperfusion, it can be divided into subtypes: warm‐dry, cold‐dry, warm‐wet, and cold‐wet.
5. Risk stratification of ventricular arrhythmias (VA): According to the existence of organic heart disease and hemodynamic abnormalities, VA can be classified into benign VA, potentially malignant VA, and malignant VA. It is reported that the risk of heart attacks increases when the load of premature ventricular contraction is more than 20%.

### 2.1 Issues

In spite of significant progress that has been made in the assessment of heart failure, it is undeniable that non‐cardiologists lack cardiovascular knowledge. Furthermore, the way of clinical thinking of cardiologists and non‐cardiologists is different. In addition, the diagnosis and treatment vary by geographic regions. Thus, there is an area for development. The multidisciplinary team consultation system can bridge the gap in the application of various clinical guidelines in different disciplines and promote the optimization of treatment strategies.

## 3 CURRENT STATUS OF STANDARDIZED MEDICATION

As the respiratory and circulatory systems interact with each other, it is worth considering whether to adopt a combined strategy to treat cardiovascular disease and pneumonia at the same time or treat pneumonia only. It might be more conductive to the treatment of pneumonia when the management is focused on the cardiovascular disease. The accumulation of experience benefits optimized treatment. The flexible conversion of strategies is helpful for improving the efficiency and success rate of treatment. Once a treatment strategy fails, it should be replaced with another one quickly, without delay. A comprehensive treatment strategy is a great advance in the treatment of comorbidity in the elderly.

The medications for COVID‐19 include antiviral therapy, such as chloroquine phosphate, lopinavir/ritonavir, and remdesivir, prophylactic use of antibiotics, glucocorticoid in severe COVID‐19, plasma antibodies, and traditional Chinese medicine. There is evidence that protecting pulmonary blood vessels and reducing airway and alveolar inflammatory secretions are the keys to treatment. continuous renal replacement therapy and artificial liver are beneficial to remove inflammatory factors and protect liver and kidney functions.

The treatment of cardiovascular disease includes anti‐myocardial ischemia, high blood pressure treatment, the control of ventricular rate of atrial fibrillation, and the control of ventricular arrhythmia. The treatment of heart failure involves the following: (a) treatment of chronic heart failure: HFrEF: diuretics (up to 2000 mL in 24 hours), beta‐blockers, angiotensin receptor‐neprilysin inhibitor, angiotensin receptor blocker (ARB)/angiotensin converting enzyme inhibitors (ACEI), spironolactone; HFmrEF and HFpEF: while the curative effect of beta‐blockers and ARB/ACEI remains unclear, spironolactone can reduce the risk of hospitalization of patients for heart failure in HFpEF; (b) the treatment of AMI pump failure: (i) pulmonary congestion (−), insufficient tissue perfusion (−): adjust oral drugs only; (ii) pulmonary congestion (+), insufficient perfusion (−): due diuresis, static drops of nitrate preparation; (iii) pulmonary congestion (−), perfusion insufficiency (+): if the heart rate is slow, increase heart rate; (iv) lung congestion (+), perfusion insufficiency (+): dopamine plus sodium nitrate static drops; (c) the treatment of acute left heart failure: in addition to the warm‐dry type to adjust oral drugs, cold‐dry type to expand capacity, cold‐wet type, such as blood pressure with vasoconstrictor and mechanical circulation support, other types can be treated with cardiotonic, diuretics, and vasodilators; (d) ventricular arrhythmia: beta blockers, amiodarone, defibrillation when needed.

### 3.1 Problems 

(a) The curative effect of antiviral drugs is still in the clinical research stage. (b) An effective vaccine is not yet available. (c) There is no completely unified dose and course of glucocorticoid use in severe patients. (d) The insufficient use of traditional Chinese medicine is an urgent problem to be solved in our country.

Regarding the ACEI/ARB controversy, it is currently believed that, with the exception of a few individuals with angioedema, the ACC guideline emphasizes that patients with cardiovascular disease should receive strict medication in accordance with the guideline to provide additional protection.

### 3.2 Medication directions for the elderly 

The total amount of liquid and excessive speed of infusion should be appropriately limited. Generally, infusion speed < 0 mL/h or <1400 mL/24 h (except for fluid resuscitation with additional capacity) has little impact on cardiac function of the elderly with cardiovascular disease. At the same time, it is necessary to guard against aspiration pneumonia and heed nutrition support.

## 4 STATUS OF CARDIOPULMONARY REHABILITATION AND SECONDARY PREVENTION

Pulmonary rehabilitation includes exercise training, patient education, and behavior change. It aims to change the physical and mental state of patients with chronic respiratory diseases, improve patients’ compliance with long‐term healthy behaviors, alleviate symptoms, and reduce disability.

Effective cardiac rehabilitation is very important to help patients improve their quality of life, and to prevent reinfarction and sudden cardiac death.^8^ Different plans are made for the acute and convalescent phases. Cardiac rehabilitation starts from admission, including acute phase (4‐7 days after onset), convalescence (7 days‐4 weeks in early convalescence, 2‐6 months in late convalescence), and maintenance phase (6 months to lifetime).^9^

Secondary prevention is the key to reducing the recurrence rate and mortality of cardiac events. The current problem is an overall decline in the use of cardiovascular medications after discharge according to long‐term follow‐up. So, it is significant for doctors to develop individualized treatment and conduct early and close follow‐up. It is necessary to note that patients with viral myocarditis may develop malignant arrhythmia or exacerbation of atrioventricular block within 2‐4 weeks of onset.

To sum up, doctors should conduct standardized diagnostic stratification and rational medication, especially in the early stage. It is worth emphasizing that the multidisciplinary team consultation system could shorten the course of treatment and reduce the occurrence of critical illness and multiple organ failure.

## CONFLICTS OF INTEREST

Nothing to disclose.
